# Presence and User Experience in a Virtual Environment under the Influence of Ethanol: An Explorative Study

**DOI:** 10.1038/s41598-018-24453-5

**Published:** 2018-04-23

**Authors:** Mario Lorenz, Jennifer Brade, Lisa Diamond, Daniel Sjölie, Marc Busch, Manfred Tscheligi, Philipp Klimant, Christoph-E. Heyde, Niels Hammer

**Affiliations:** 1 Chemnitz University of Technology, Professorship for Machine Tools and Forming Technology, Reichenhainer Straße 70, 09126 Chemnitz, Germany; 2 University Clinics of Leipzig, Department of Orthopedics, Trauma and Plastic Surgery, Liebigstraße 20, 04103 Leipzig, Germany; 3 Austrian Institute of Technology GmbH, Innovation Systems, Donau-City-Str. 1, 1220 Vienna, Austria; 4 University of Gothenburg, Department of Applied Information Technology, Division of Interaction Design, SE-412 96 Gothenburg, Sweden; 5 handcheque GmbH, c/o weXelerate, Praterstraße 1, 1020 Vienna, Austria; 6 University of Otago, Department of Anatomy, Clinical Anatomy Research Group, 270 Great King Street, Dunedin, 9054 New Zealand; 7 University of Leipzig, Department of Anatomy, Liebigstraße 13, 04103 Leipzig, Germany

## Abstract

Virtual Reality (VR) is used for a variety of applications ranging from entertainment to psychological medicine. VR has been demonstrated to influence higher order cognitive functions and cortical plasticity, with implications on phobia and stroke treatment. An integral part for successful VR is a high sense of presence – a feeling of ‘being there’ in the virtual scenario. The underlying cognitive and perceptive functions causing presence in VR scenarios are however not completely known. It is evident that the brain function is influenced by drugs, such as ethanol, potentially confounding cortical plasticity, also in VR. As ethanol is ubiquitous and forms part of daily life, understanding the effects of ethanol on presence and user experience, the attitudes and emotions about using VR applications, is important. This exploratory study aims at contributing towards an understanding of how low-dose ethanol intake influences presence, user experience and their relationship in a validated VR context. It was found that low-level ethanol consumption did influence presence and user experience, but on a minimal level. In contrast, correlations between presence and user experience were strongly influenced by low-dose ethanol. Ethanol consumption may consequently alter cognitive and perceptive functions related to the connections between presence and user experience.

## Introduction

Virtual Reality (VR) has been used for a variety of applications for the last few decades, ranging from entertainment via medical applications to industrial use. In the biomedical context, VR is applied to treat different types of mental health disorders e.g. fear of spiders, heights, public speaking, schizophrenia or substance disorders^1,2^. VR is also applied successfully in the rehabilitation of stroke patients^3^ or for the treatment of post-traumatic stress disorders (PTSD)^4^. Positive effects in patient treatment have been demonstrated^3–5^, proving VR to be capable of successfully influencing behavior on a subconscious level. Associated with the experience of a virtual scenario as being real is a high sense of presence^6^ – the feeling of ‘being there’.

According to Slater and Wilbur, there are basically two main pillars to support presence, context and immersion^6^. Context indicates how convincing the setting and the storytelling of the virtual scenario are for the user. Immersion focusses on the technical aspects to provide sufficient sensory information for the perception system, e.g. good stereoscopic imaging with high resolution images and an appropriate framerate^7^.

In general, user experience is important for all applications as it describes ‘a person’s perceptions and responses that result from the use and/or the anticipated use of a product, system or service’^8^. A good user experience enables the user to quickly learn how an application is operated, to operate it efficiently and to ‘enjoy’ the use of the application. Therefore, user experience is a measure of the user’s willingness to utilize a VR application and their emotional connection with it^9^. This is an important aspect for all applications, e.g. for the above-mentioned applications in the biomedical and psychological domains, as in our case. In a medical context, the importance of a good user experience may be exemplified by the way in which patients adapt to VR applications, and the underlying motivation, which could be partially driven by the joy of using such applications, quickly submerging into it.

The rationale for a high presence to be desired for most VR application is that the user should behave and react the same way in the virtual scenario as in reality. The user’s feeling to be present in a virtual scenario forms the basis for almost all VR applications, as it enables them to be involved. VR applications for the treatment of phobias^2^ is one example. However, in more research areas, VR is used in studies as a test environment, because it can provide a higher ecological validity (realism) than a laboratory environment, whilst at the same time it allows for a higher control than in a real environment, e.g. to assess the user experience of a product in an early phase of development^10–14^. The basic assumption of such studies is often that the participants would behave and react in the same way as in a real situation. However, this assumption is corrupted, as we demonstrated that presence has an influence on user experience (Brade *et al*.^15^, Busch *et al*.^16^). In an earlier work on VR^15^ we could show that presence, user experience and usability were significantly different between the real world and the VR measurements. Furthermore we could show a strong connection between presence with user experience and usability in the VR environment, which was absent in the real world.

Both presence and user experience are connected to perception, cognition and emotion. The first step to achieve presence in a VR scenario is to allow the users to perceive the computer generated virtual world via their perceptional system. This artificial sensory input has to be cognitively processed by the brain. Depending on the quality of the sensory input and its cognitive processing, a weaker or stronger feeling of ‘being there’ in the VR scenario by the users is achieved. This results in a perceived degree of presence. The engagement of users in a VR scenario can trigger any emotion depending on the content, e.g. fear as in VR phobia treatment applications^2^. However, the sole factor of being present in VR can trigger emotions like excitement or even discomfort resulting from negative effects, such as cybersickness^17^.

However, the exact understanding of the term ‘presence’ and processes of perception and cognition, which lead to the formation of presence, are debated since the early 1990s. Coelho *et al*.^18^ summarized the different interpretations and categorized them into media-presence, as a result caused by technology to be in VR, and inner presence, the general feeling of being present e.g. in reality, dreams, books, movies, VR etc^18^. This separation has also prevailed in recent research described by Diemer *et al*.^19^. Further, Slater^20^ stated that presence is actually binary (present or not present) and should not be mixed with emotions and involvement^20,21^. However, existing presence questionnaires often reflect that separation by providing scales for spatial presence and involvement, but are still considering all scales as presence^22–24^. Most literature does not follow Slater’s^20^ harsh separation and implicitly argues for a tight interrelations of presence, involvement and emotion^18,19^. We also follow the view of Coelhos *et al*.^18^ on inner presence as “an experience common among different types of human experiences independent of any technology” a “neuro psychological phenomenon”^18^. To emphasize this, Coelho *et al*.^18^ relate to the work of Loomis^25^ who considers a synthetic experience, like in VR, in line with the normal every day experience of the real world. The world as we perceive it, results from our senses and nervous system interacting with the physical world^25^. Moreover “the physical world, including our nervous system, is not given to us directly through experience but is inferred through observation and critical reasoning”^25^. Also the more recent research conducted by Seth *et al*.^26^ argues in this direction as they consider presence “a basic property of normal conscious experience”, occurring constantly. Seth *et al*.^26^ provide the example of schizophrenia and depersonalization disorder for a disturbance in presence in normal reality^26^. Following this theory, in VR, the initial sensory input is mediated by technology but once perceived by our senses undergoes the same interpretation mechanism. Coelho *et al*.^18^ further note that the perceived control over a VR experience, and the possibility to interact, can lead to the user forgetting the technology and being present. The extent of control and the quality of the sensory input created by technology (immersion) can then lead to a different degree of feeling present in VR^18,27^. This important role that immersion plays in the creation of presence is further investigated by Diemer *et al*.^19^, but they also emphasize the importance of emotion for the creation of presence. They rest on the theory of Seth *et al*.^26^ who postulate that presence, in general always, results from the mismatch of the actual current emotional state of an individual and a predicted emotional state resulting from experience. In terms of VR this is the essential suspension of disbelief, necessary for an individual to feel present in a VR scenario, despite knowing that it is located in a different place in the real world. Following this direction, and evaluating different research regarding the effect of emotions on presence, Diemer *et al*.^19^ developed an interoceptive attribution model of presence^19^. According to that model the reported presence of individuals through presence questionnaires is the result of a cognitive judgment from the immersiveness, the interactivity provided by the VR system and the emotional arousal from the perceived content of the VR scenario^19^. The described functions that the sensory input (immersion) and emotion play in the formation of presence in the real world and in VR are in line with the different presence findings in our previous study^15^. There, the differences in the evoked emotions and the sensory input between the real world and the virtual environment could explain the difference in presence.

The connection of user experience with perception, cognition and emotion may be more obvious. An application must stimulate the sensory cues of the users so that they can use it. The process of learning how to use an application is a cognitive task. The experience of the application will lead to an emotional attitude of the users towards it. Our previous studies further suggest that there is a connection between presence and user experience^15,16^.

Human perception, cognition and emotion can be influenced by drugs such as ethanol, codeine, amphetamines, tranquilizers or other similar substances, which could potentially be confounders. Ethanol may influence perception of contrasts, cause blurry vision, decision making, ease trust in others and affect emotion^28–32^.

These effects are well studied in the context of drunk driving studies. VR is commonly applied as a setting for such studies, instead of real and potentially dangerous driving scenarios. However, most VR drunk driving studies failed to assess presence and the influence of ethanol, which may negatively influence the validity of the results. Furthermore, now that VR is on the verge of becoming a technology for mass entertainment and that major international companies are investing billions of dollars in the consumer branch of VR, the effect of ethanol on a VR experience becomes relevant^33–35^. It is therefore important to gain an understanding of how ethanol intake affects presence to define key variables for VR industries and consumers alike. This question can be further abstracted as to what influence drugs have on presence in general and especially in medical VR applications. Such applications could use VR for surgical training as well as phobia or PTSD treatment, especially as alcohol dependent patients often suffer from a comorbid phobic disorder^36–38^. These VR scenarios might involve some level of drug influence, either on the patient using VR or on the physician, e.g. influenced by narcotic gases or drugs evaporating from the patient.

An exploratory research into the effects of ethanol on presence does consequently have high relevance, as it is widely consumed, accepted in most societies and easy to access^35^. Therefore, it is a potential confounder for presence and user experience in most VR applications. To date, to the authors’ best knowledge, there is no baseline data on how ethanol could influence presence and user experience, and how it may influence their association in VR. This given exploratory study aims at contributing towards a basic understanding of this complex topic. Using a similar study setting as in our previous study, researching presence and its connection to user experience^15^, this study addresses the following hypotheses:

- H1: Ethanol consumption will affect presence and user experience in VR, due to altered perception, cognition and emotion.
- H2: Ethanol consumption will influence the association between presence and user experience in VR due to altered perception, cognition and emotion.
- H3: The kinetics of ethanol breakdown influences presence and user experience in VR, with different effects on presence and user experience in those metabolizing ethanol quicker than the median in a representative cohort.

## Methods

### Study Setup

The given hypotheses were tested in a prospective exploratory study design with a retrospective control group. Ethanol consumption was defined as an independent variable and 11 dependent variables for presence, usability and user experience for the participants of the exploratory arm. Institutional approval was obtained from the Institute for Machine Tools and Production Processes of the Chemnitz University of Technology, and ethical approval was obtained from the University of Leipzig (number: 251/17-ek). All participants provided written and informed consent. The experiments were conducted according to the principles of the Declaration of Helsinki.

**Figure 1:**
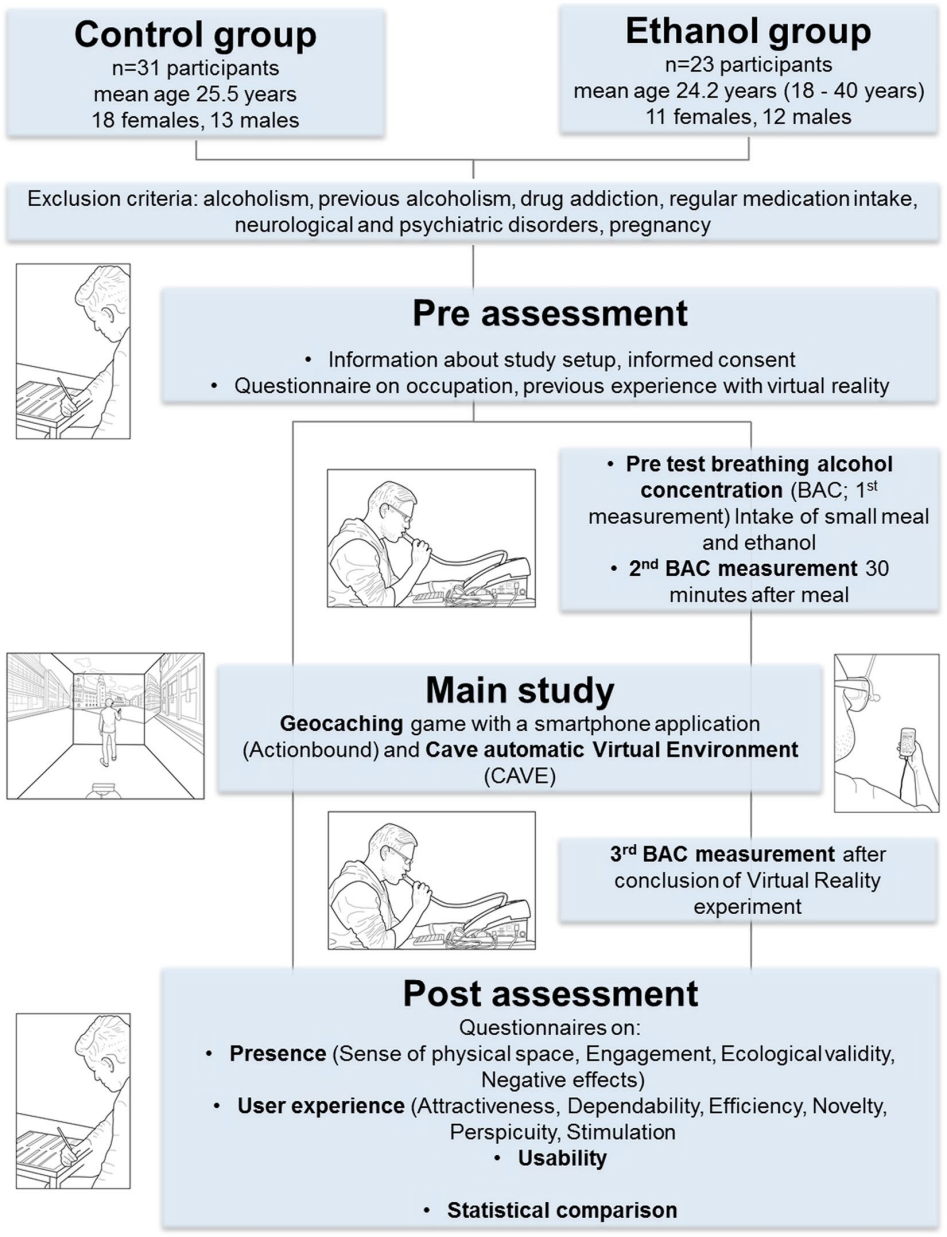
Graphical representation of the study procedure (Drawings by Robbie McPhee).

For the non-ethanol condition, data from a previously published study, investigating the effects of presence on usability and user experience in virtual and real environments, was used^15^. This study used a combined virtual scenario and a real geocaching experiment to navigate in the city center of Chemnitz, Germany. The control group was representative of an average sample of the general population. To ensure comparability between the control group and the ethanol group, the study protocol from the previously published study^15^ was adapted to include ethanol consumption and breath ethanol measurements. The protocol consisted of three parts: (1) pre-assessment, (2) main study and (3) post-assessment (see Fig. 1). All demographic variables (age, gender and education) were obtained, as well as a self-assessment on the participants’ abilities to navigate with digital and paper-based maps, and their familiarity with geocaching and previous VR experience. The adaptation in the study protocol only concerned the pre- and the post-assessment; the main part of the study was conducted in exactly the same way. The pre-assessment was altered from the previous protocol in a way that the participants performed an initial ethanol measurement, followed by the intake of a small meal (half a bread roll with cheese) and the ethanol mixed with orange lemonade, and a waiting time of 30 minutes followed by a second ethanol measurement. The post-assessment was only adapted to include an ethanol measurement right after the participants finished the main study and before they filled out the questionnaires. To ensure comparability between the study and the control group, the demographics questionnaire was extended by two items; surveying the frequency and the amount of alcohol intake by the participants. Baseline data from the previously published study^15^ was conducted three months prior to the acquisition of the data in this present study.

**Figure 2:**
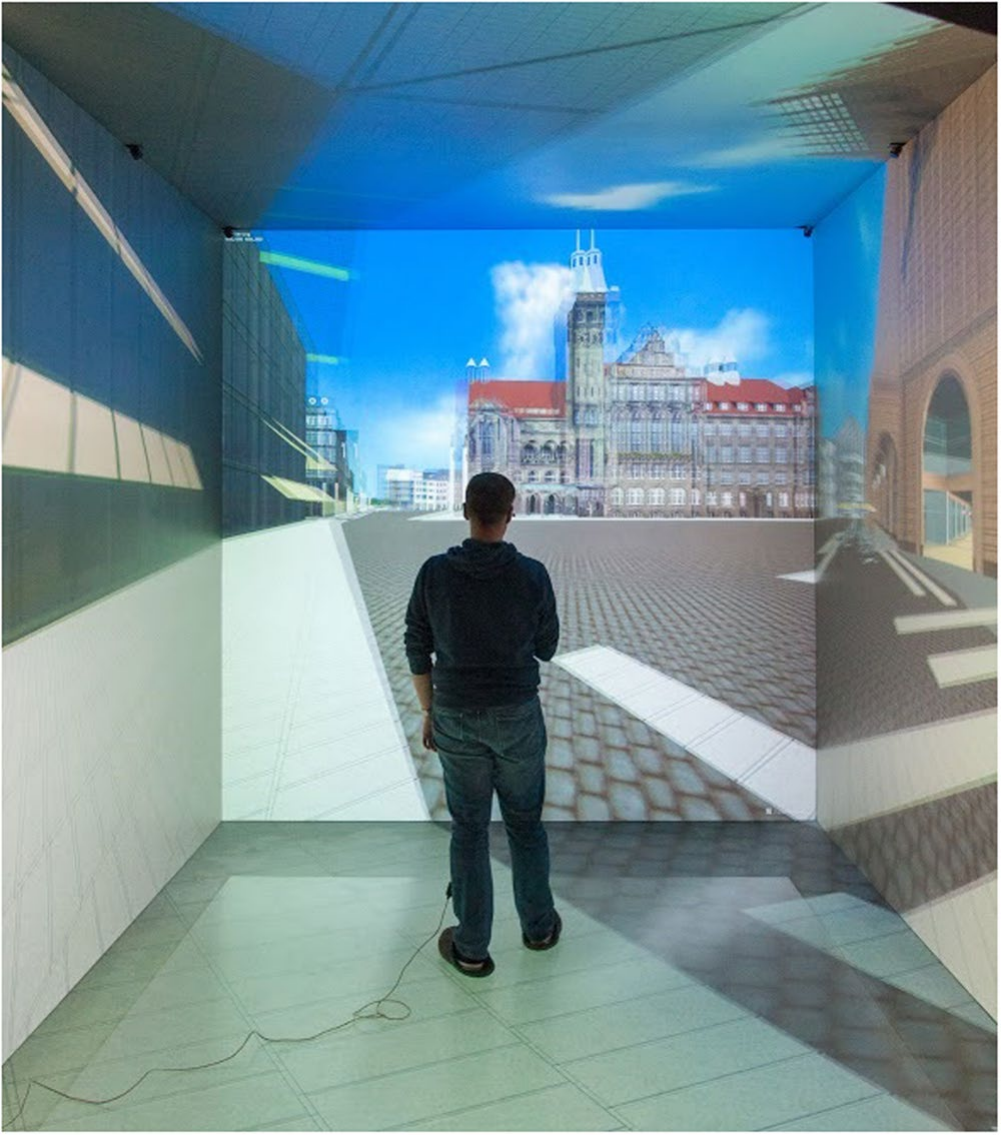
The virtual environment (CAVE) showing the city center of Chemnitz, Germany.

In the main study the participants performed a geocaching game with a smartphone application in the virtual city center of Chemnitz, Germany. The Actionbound application from Simon Zwick and Jonathan Rauprich GbR^39^ was used for the geocaching game, consisting of a tour of seven locations with a total length of 1.7 km. The participants used a digital map displaying the city center, their present position, and the location of the next point, to help them find the sought-out location. Every participant was asked to solve the same tasks in the same sequence. The experiment took place in a five-sided CAVE (Cave automatic Virtual Environment) at the Virtual Reality Centre Production Engineering, located at the Chemnitz University of Technology, Germany (Fig. 2). The employed CAVE is built on the principles of Cruz-Neira *et al*.^40,41^ with an edge length of three meters. A cluster of 11 computers equipped with NVidia Quadro 6000 graphic cards and 20 full HD projectors handled the rear-projection of the images on the sides of the cube. To achieve stereoscopic vision, passive circular polarization was used. An optical infrared tracking system by ART GmbH (Weilheim i. OB., Germany) with six cameras was used to track the orientations and position of the participants’ heads to calculate the respective point of view in the virtual world. The tracking of the smartphone in the virtual city center was simulated with an artificial GPS signal. For locomotion in the virtual environment we used a gesture based navigation system developed by Lorenz *et al*.^42^ that uses a Microsoft Kinect body tracking system. It recognizes the participants’ skeletons filmed from behind. The usage of this locomotion method in previous studies showed, that for some people the tracking was less stable and that some had more difficulty controlling their movements in the VR scenario than others. But the vast majority considered it a good method for locomotion^42,43^. Following the main part, the dependent variables were obtained in the post-assessment with post-test questionnaires.

### Recruitment

The participants for the ethanol group were recruited using social media. Inclusion criteria was the occasional to regular consumption of ethanol in social contexts, excluding alcoholics, sober alcoholics, people with psychiatric or neurological disorders, people who regularly medicated or had any health grounds dissuading them from the consumption of ethanol, and were not suspected to be pregnant. The demographic variables such as age, sex and educational background were matched to the control group. The participants of the non-ethanol group were recruited using social media and the mailing lists of the faculties of the Chemnitz University of Technology. Inclusion criteria was age between 18 and 40. The principal investigators asked the participants prior to the study about their last ethanol consumption and paid attention to them displaying no sign of ethanol or any other drug intoxication.

### Participant sample

Twenty-three participants took part in the experiment for the ethanol group. The sample size of the control group was 31. Table 1 gives an overview of the participants’ distribution, their gender and professional occupation. Table 2 shows the distribution of the age of the participants, the results of their self-assessment in the ability to read a map, and the distribution of their previous contact with virtual reality systems and geocaching, for both the control and ethanol groups. The *P*-values of a Mann-Whitney-U-test are also presented, showing that there were no differences between the groups.

**Table 1:** Distribution of participant gender and professional occupation for the control and ethanol groups.

	Control Group	Ethanol Group
Gender	Female: 18 (58%)	Female: 11 (48%)
	Male: 13 (42%)	Male: 12 (52%)
Professional occupation	Students: 24 (77%)	Students: 10 (44%)
	Other: 7 (23%)	Other: 13 (56%)

**Table 2:** Distribution of participant age, results of the self-assessment in ability to read a map and distribution of previous contact with virtual reality systems (CAVE) and geocaching, for the control and ethanol groups including *P*-values (Mann-Whitney-U) testing for differences between the groups

Group	P-value	Control Group	Ethanol Group
Age (mean)	0.79	25.5	24.2
		(SD = 7.2)	(SD = 3.9)
Ability to read a map (paper)	0.73	excellent = 12 (39%)	excellent = 7 (30%)
		good = 15 (49%)	good = 10 (45%)
Ability to read a map (mobile)	0.60	excellent = 9 (29%)	excellent = 5 (22%)
		good = 18 (58%)	good = 13 (57%)
Previous contact with VR-systems (yes)	0.73	8 (26%)	5 (22%)
Previous contact with geocaching (yes)	0.07	11 (35%)	3 (13%)

### Independent Variable: Ethanol Consumption

**Figure 3:**
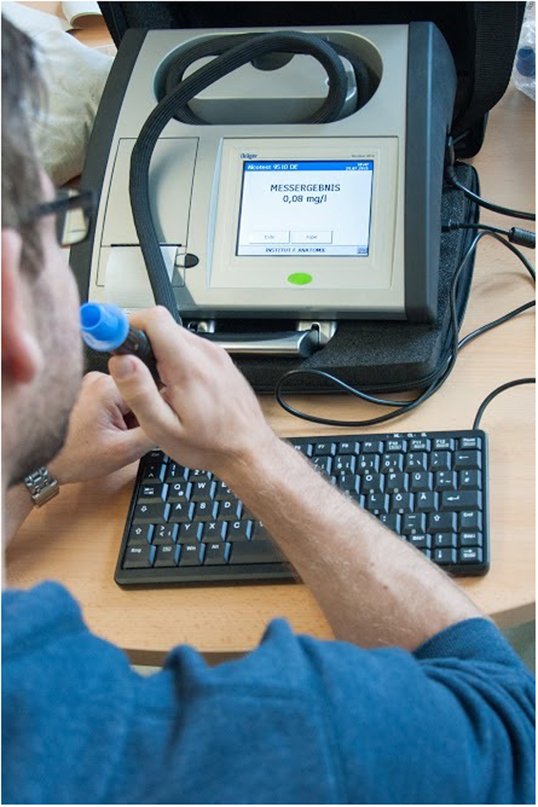
Measurement of the ethanol level with the Alcotest 9510 (Dräger, Lübeck, Germany).

Following initial information about the nature of the research the participants completed the pre-assessment, including the first measurement of the ethanol level. This measurement was taken to ensure that the participants were sober prior to the experiment. The measurement was carried out with the Dräger Alcotest 9510 (Fig. 3), the only device that is presently admissible as evidence in legal proceedings. The Alcotest 9510 has a measuring range of 0–3 mg/l and a standard deviation of < 0.006 mg/l, thereby providing sufficient accuracy. Every measurement took five minutes and consisted of two measuring processes from which the values were averaged. The time and values of the measurements were recorded in the study protocol. Following this the participant ate a small meal – half a bread roll with cheese, followed by the ethanol mixed with orange lemonade. As we did not want to exceed a blood alcohol concentration (BAC) of 0.4‰, we calculated the quantity of ethanol based on height, weight, age and gender of the participant. For this purpose the Widmark formula, with Watson’s extension^44^, was used. Two further measurements were taken, one 30 minutes after the drink and the last one after finishing the CAVE experiment, approximately 20 minutes later. Lastly, the participants filled out the post-assessment questionnaires.

### Measure: presence

The International Test Commission – Sense of Presence Inventory (ITC-SOPI) by Lessiter *et al*.^23^ was used for evaluating presence. The ITC-SOPI rating scale is made up of 44 items and four factors. The four factors are:

#### Ecological validity

Describes the users feelings of the believability, realism and naturalness of the displayed environment (“The content seemed believable to me”)^23^.

#### Engagement

Describes the interest and the involvement of the users in the displayed environment, their interest in the content and their general joy of the VR experience (“I felt myself being drawn in”)^23^.

#### Negative effects

Describes all negative effects in occurrence with the VR experience, e.g. nausea, mild dizziness or kinetosis (“I felt disorientated”)^23^.

#### Sense of physical space

Describes the users feelings of physically being within the displayed environment and the feeling to interact and control objects (“I felt as though I was participating in the environment”)^23^.

A short version of the ITC-SOPI with only the three top loading items per scale had been used as shown previously^15^ for the control group. The rating of each statement was made on a five-point Likert scale.

### Measure: user experience

User experience^45^ is defined as to how users subjectively assess a product, combining usability factors such as efficiency, dependability, fault tolerance, learnability and effectiveness with aesthetics, joy-of-use and attractiveness^46^. For measuring user experience the validated user experience questionnaire (UEQ) by Laugwitz *et al*.^47^ was used. The questionnaire included 26 bipolar items divided into 6 scales:

#### Attractiveness

Describes the users general impression of the product^46^.

#### Dependability

Describes the users feeling if the interaction with the product was easy and predictable and the feeling of having control over the interaction^46^.

#### Efficiency

Describes how quickly and efficiently the user could operate the product^46^.

#### Perspicuity

Describes how easily the user could understand the product^46^.

#### Novelty

Describes whether the design of the product was perceived innovative, creative and aroused the attention of the users^46^.

#### Stimulation

Describes the users interest and excitement of the product and their interest to continuously use the product^46^.

The scales efficiency, dependability and perspicuity describe the pragmatic quality of the product, whereas the scales novelty and stimulation relate to its hedonic qualities. The scale attractiveness represents a positive or negative attitude to the product. Participants were asked to rate the items using a seven-point semantic differential. UEQ results had been utilized from previous data as the measure of the user experience for the control group^15^.

### Measure: usability

How the user assesses the fitness of use of a product is described by the term ‘usability’, which also represents the pragmatic aspect of user experience. To measure usability, the systems usability scale (SUS) by Brook^48^ was used, in accordance with the control group^15^. The SUS consisted of ten items that were rated on a Likert scale ranging from zero to four.

### Statistical Methods

To compare the results between the conditions, a Mann-Whitney-U or Student’s t-test was used, depending on whether the samples showed a normal distribution, as indicated by the Kolmogorov-Smirnov-test. The effect sizes for the differences of means were calculated using the Z-scores of the Mann-Whitney-U-test as described by Fritz *et al*.^49^. The two-tailed Spearman test was used to compute correlations of factors in both the ethanol and control groups. The average ethanol breakdown per hour of each participant was calculated from their second and third ethanol measurements and the time between both measures. The median was used to subdivide the sample into fast and slow ethanol metabolizer groups. Those participants who had a breakdown rate above the median were considered fast metabolizers, and those participants who had an equal or below the median, slow metabolizers.

### Data availability

The datasets generated during and/or analyzed during the current study are available from the corresponding author on reasonable request.

## Results

### Ethanol consumption characteristics

Table 3 summarizes the distribution for participant frequency of ethanol consumption. The assessment of drinking behavior showed that 10 (44%) of the participants did not consume ethanol without a social occasion. Whereas within a social occasion, 14 (62%) of the participants consumed up to four glasses of ethanol-based drinks (Table 4). One glass of ethanol equals 0.33 l beer, 0.25 l wine/sparkling wine or 0.02 l spirits. None of the participants identified themselves as addicted to ethanol, nor was there evidence from the psychological assessment regarding alcoholism in either of the groups. Consequently, statistical comparison yielded no significant differences between the groups (*P* = 1.00).

**Table 3:** Distribution of participant frequency of ethanol consumption within the ethanol group.

	Frequency of ethanol consumption
2–4 times a month	13 (57%)
2–3 times a week	6 (26%)
4 or more a week	4 (17%)

**Table 4:** Distribution of participant quantity of ethanol consumption within a social occasion (indication in glasses) within the ethanol group.

Glasses	Ethanol consumption with social occasion
1–2	4 (17%)
3–4	10 (45%)
5–6	4 (17%)
7–8	4 (17%)
9 or more	1 (4%)

Prior to the experiment all of the participants’ breath ethanol concentration measurements showed 0.00‰. Further to this no other signs of drug influence were identified. Thirty minutes after the ethanol intake the participants reached an average of 0.11 mg/l (SD = 0.03), with a 95% confidence interval of 0.09 to 0.13, corresponding to a BAC of 0.23‰ (conversion factor 2.1)^50^. An ethanol intake-related increase in the ethanol measures was observed in all participants. In the third measurement, after completing the experiment, the mean breath alcohol level was 0.06 mg/l (SD = 0.03) equating to a BAC of 0.13‰. The ethanol intake related BAC did in not exceed 0.4‰ in any of the participants during the experiments.

### Psychometric properties of the scales

The Cronbachs’ alpha values for the scales of the ITC-SOPI, SUS and UEQ reached values of 0.50 or more with the only exception being the efficiency factor from the UEQ. The efficiency factor from the UEQ was therefore excluded from further analyses, but will be listed for the sake of completeness. All Cronbach’s alpha values can be found in the supplement files.

### H1: Influence of ethanol intake on presence, usability and user experience

**Figure 4:**
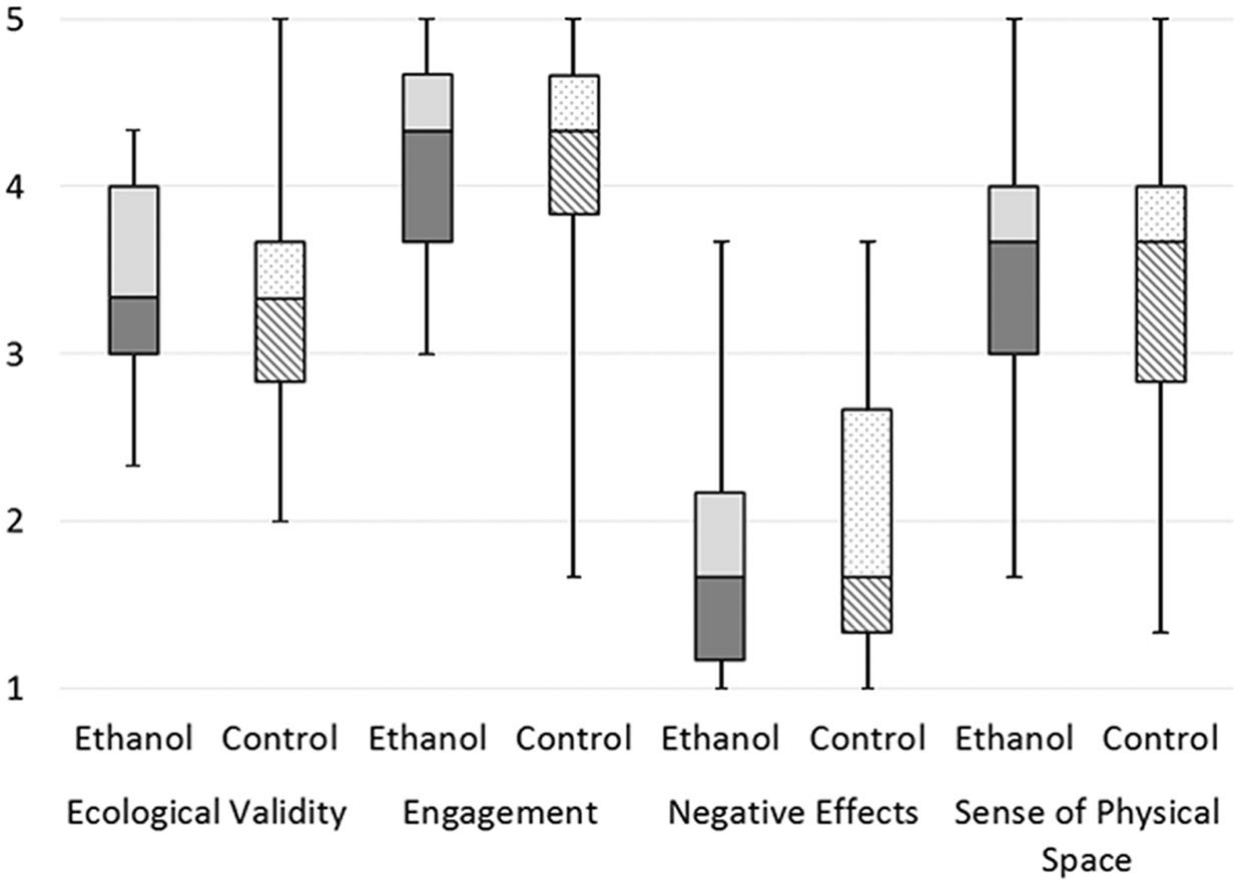
Boxplot of the presence factors for the ethanol group and for the control group. Whiskers indicate the 25^th^ and 75^th^ percentiles.

**Figure 5:**
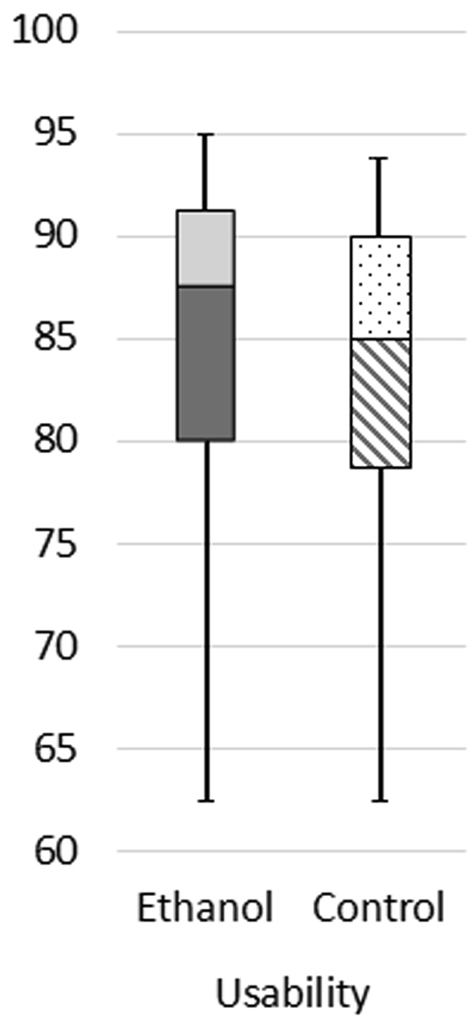
Boxplot of the usability for the ethanol group and for the control group. Whiskers indicate the 25^th^ and 75^th^ percentiles.

**Figure 6:**
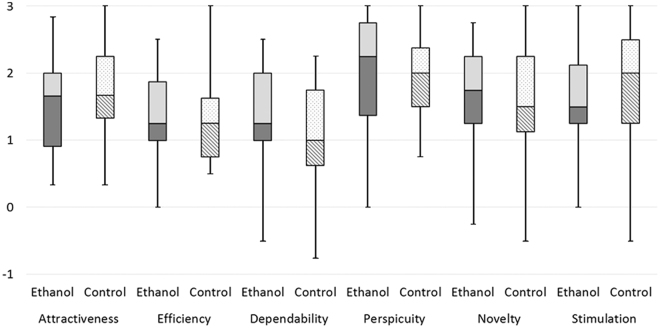
Boxplot showing user experience factors for the ethanol group and for the control group. Whiskers indicate the 25^th^ and 75^th^ percentiles.

Table 5 shows the mean values, standard deviations and medians of all factors for presence and user experience along with the associated *P*-values and η²-values for the effect size. They showed no significant differences between the ethanol and the control groups for any factor. Also the effect sizes showed no or only a small effect^51^. Figure 4 shows boxplots for the presence factors of the ethanol and control groups, Fig. 5 for usability and Fig. 6 for the user experience factors.

**Table 5:** Means, standard deviations (first row) and medians (second row) of the presence, usability and user experience factors for the control and ethanol groups with their *P*-values (Mann-Whitney-U) for significance testing and η²-values for effect sizes.

	P-value	η²-value	Control Group	Ethanol Group
Ecological validity	0.39	0.014	3.35 (SD = 0.69)	3.48 (SD = 0.60)
			3.33	3.33
Engagement	0.56	0.006	4.15 (SD = 0.73)	4.11 (SD = 0.57)
			4.33	4.33
Negative effects	0.27	0.023	2.09 (SD = 0.86)	1.83 (SD = 0.80)
			1.67	1.67
Sense of physical space	0.57	0.006	3.34 (SD = 0.89)	3.51 (SD = 0.80)
			3.67	3.67
Usability	0.60	0.005	82.98 (SD = 9.23)	84.23 (SD = 8.71)
			85.00	87.50
Attractiveness	0.41	0.013	1.73 (SD = 0.75)	1.54 (SD = 0.74)
			1.67	1.67
Dependability	0.13	0.042	1.05 (SD = 0.76)	1.35 (SD = 0.80)
			1.00	1.25
Efficiency	0.24	0.026	1.28 (SD = 0.61)	1.40 (SD = 0.62)
			1.25	1.25
Perspicuity	0.92	<0.001	2.01 (SD = 0.66)	1.93 (SD = 0.91)
			2.00	2.25
Novelty	0.76	0.002	1.62 (SD = 0.86)	1.62 (SD = 0.84)
			1.50	1.75
Stimulation	0.24	0.026	1.82 (SD = 0.91)	1.60 (SD = 0.73)
			2.00	1.50

### H2: Influence of ethanol consumption on the connection between presence, usability and user experience

Tables 6 and 7 show the correlations of the presence factors with the user experience factors for the ethanol and the control groups. In the control group 18 of 28 correlations were found to be at a significant level, whilst in the ethanol group only seven correlations were observed. The calculation of partial correlations did not give adequate results, as a graphical analysis showed that the correlations were strongly nonlinear.

**Table 6:** User experience factors and usability correlated (Spearman, two-tailed) with presence factors (“Ecological validity” and “Engagement”) for the ethanol and control groups. With *P-*values (bold fields are significant).

	Ecological validity		Engagement	
	Control Group	Ethanol Group	Control Group	Ethanol Group
Attractiveness	**0.61 (** ***P*** ** < 0.001)**	**0.53 (** ***P*** ** = 0.009)**	**0.84 (** ***P*** ** < 0.001)**	0.37 (*P* = 0.08)
Dependability	**0.66 (** ***P*** ** < 0.001)**	0.36 (*P* = 0.09)	0.28 (*P* = 0.13)	0.04 (*P* = 0.87)
Efficiency	0.32 (*P* = 0.08)	0.32 (*P* = 0.14)	**0.46 (** ***P*** ** = 0.009)**	0.001 (*P* = 1.00)
Perspicuity	**0.49 (** ***P*** ** = 0.005)**	0.27 (*P* = 0.22)	**0.42 (** ***P*** ** = 0.02)**	0.34 (*P* = 0.12)
Novelty	0.28 (*P* = 0.12)	**0.41 (** ***P*** ** = 0.05)**	0.31 (*P* = 0.09)	0.28 (*P* = 0.20)
Stimulation	**0.45 (** ***P*** ** = 0.01)**	−0.03 (*P* = 0.88)	**0.46 (** ***P*** ** = 0.009)**	0.06 (*P* = 0.80)
Usability	**0.42 (** ***P*** ** = 0.02)**	0.19 (*P* = 0.38)	**0.57 (** ***P*** ** = 0.002)**	**0.42 (** ***P*** ** = 0.04)**

**Table 7:** User experience factors and usability correlated (Spearman, two-tailed) with presence factors “Negative effects” and “Sense of physical space”) for the ethanol and control groups. With *P-*values (bold fields are significant).

	Negative effects		Sense of physical space	
	Control Group	Ethanol Group	Control Group	Ethanol Group
Attractiveness	−0.14 (*P* = 0.47)	−0.13 (*P* = 0.54)	**0.65 (** ***P*** ** < 0.001)**	**0.52 (** ***P*** ** = 0.01)**
Dependability	**−0.49** (***P* = 0.005)**	−0.32 (*P* = 0.13)	0.32 (*P* = 0.08)	0.31 (*P* = 0.16)
Efficiency	−0.11 (*P* = 0.57)	−0.27 (*P* = 0.22)	**0.44 (** ***P*** ** = 0.01)**	0.25 (*P* = 0.25)
Perspicuity	**−0.36 (** ***P*** ** = 0.048)**	**−0.68 (** ***P*** ** < 0.001)**	0.32 (*P* = 0.08)	0.18 (*P* = 0.41)
Novelty	0.20 (*P* = 0.28)	−0.20 (*P* = 0.37)	**0.42 (** ***P*** ** = 0.02)**	**0.58 (** ***P*** ** = 0.004)**
Stimulation	−0.09 (*P* = 0.65)	−0.38 (*P* = 0.07)	**0.49 (** ***P*** ** = 0.005)**	0.21 (*P* = 0.33)
Usability	**−0.50 (** ***P*** ** = 0.004)**	**−0.72 (** ***P*** ** < 0.001)**	**0.36 (** ***P*** ** = 0.045)**	0.27 (*P* = 0.21)

### H3: Influence of ethanol metabolism on presence, usability and user experience

The median for the ethanol breakdown of the participants was 0.11 mg/l (0.23‰.) with 11 participants in the fast metabolizers group and 12 participants in the slow metabolizers group. The median, as a statistical criterion, was chosen for two reasons. First, a formal average ethanol breakdown rate does not exist. As a rule of thumb 0.16‰ is often used as the average ethanol breakdown rate. However, even within an individual the average breakdown rate can change depending on various factors^50^. Moreover, as Jones^50^ showed there is a corridor ranging from 0.1‰ to 0.25‰, with either one of these values used in legal-forensic questions, e.g. to determine if a suspect following a crime could have been drunk and incapacitated, or vice versa if a suspect claiming to have consumed a minute quantity of ethanol following a conviction comes up with a reasonable explanation^52^. Second, by splitting the ethanol group using the median results into two groups containing 12 and 11 samples respectively. These group sizes justified the use of a test for significant differences. By using the rule of thumb ethanol break down rate of 0.16‰ group sizes of N = 15 for the fast metabolizers and N = 8 slow metabolizers would result, therefore not justifying a significant test.

Table 8 shows the mean values, standard deviations and median of the fast and slow metabolizers. Significant differences could be found between the fast and slow metabolizers in the presence factor (negative effects) and in the user experience factor (perspicuity), both showing large effects.

**Table 8:** Means, standard deviations (first row) and medians (second row) of the presence, usability and user experience factors for the fast and slow metabolizers and their *P*-values (Mann-Whitney-U) for significance testing (bold fields are significant) and η²-values for effect size.

	P-value	η²-value	Slow metabolizers	Fast metabolizers
Ecological validity	1.00	<0.001	3.47 (SD = 0.63)	3.48 (SD = 0.60)
			3.50	3.33
Engagement	0.52	0.019	4.03 (SD = 0.64)	4.21 (SD = 0.50)
			4.00	4.33
**Negative effects**	**0.049**	**0.170**	**2.19 (SD = 0.93)**	**1.42 (SD = 0.40)**
			**2.00**	**1.33**
Sense of physical space	0.77	0.004	3.39 (SD = 0.99)	3.64 (SD = 0.55)
			3.67	3.67
Usability	0.57	0.015	82.71 (SD = 9.97)	85.91 (SD = 7.18)
			83.75	87.50
Attractiveness	0.87	0.001	1.50 (SD = 0.70)	1.58 (SD = 0.82)
			1.58	1.67
Dependability	0.25	0.060	1.15 (SD = 0.81)	1.57 (SD = 0.78)
			1.25	1.75
Efficiency	0.29	0.052	1.23 (SD = 0.69)	1.57 (SD = 0.50)
			1.25	1.50
**Perspicuity**	**0.009**	**0.284**	**1.48 (SD = 0.95)**	**2.43 (SD = 0.57)**
			**1.50**	**2.25**
Novelty	1.00	<0.001	1.63 (SD = 0.86)	1.61 (SD = 0.87)
			1.75	1.75
Stimulation	0.46	0.026	1.44 (SD = 0.81)	1.77 (SD = 0.62)
			1.50	1.75

### Summary of the results

The given results of this study have shown that no significant difference between the ethanol and control group could be detected for any of the factors of the ITC-SOPI, SUS and UEQ questionnaire (Table 5). It was further found that seven of the 28 factors correlated significantly in the ethanol group, in contrast to 18/28 factors in the control group (Tables 6 and 7). These differences in the number of significant correlations between both groups were particularly strong for the presence factors ‘Sense of physical space (2 vs. 5)’, ‘Engagement (1 vs. 5)’ and ‘Ecological validity (2 vs. 5)’. For the ‘Negative effects’ there was only a difference of 2 vs. 3 (Tables 6 and 7). The results for the comparison of fast vs. slow ethanol metabolizers revealed significant differences for the factors ‘Perspicuity’ and ‘Negative effects’ (Table 8).

## Discussion

This VR-based study determined, for the first time, the influence of low-level ethanol consumption on presence and user experience.

Being present in a virtual environment can have a number of interesting physiological and psychological effects, as the phenomenon of simulator sickness impressively shows^6,53^. The same is also said for the use of VR to treat phobias and cerebral stroke^3–5^. The phenomenon of body ownership in a virtual avatar^54,55^ or even an induced higher dissociation^56^ are fascinating examples that prove the strong physical and psychological effects VR can have on a person. Though the deeper perceptive and cognitive processes explaining the aforementioned examples and underlying phenomena, are still subject of ongoing research.

One approach which may potentially contribute to identify underlying neural mechanisms in the formation of presence are the acute and long-term effects of ethanol on the physiological and neural pathways of the brain^28–32^. A number of studies have investigated the alterations in functional connectivity^57–59^, with effects on perception, motor control, memory and cognitive performance. Anatomical regions related to the changes in these areas are the thalamus^58^, the superior frontal gyrus, cerebellum, hippocampal gyrus, basal ganglia, right internal capsule^59^, the posterior cingulate cortex and the precentral gyrus with the sensorimotor network^57^. Kleinloog *et al*. have shown that the posterior cingulate cortex plays a vital role in visuospatial evaluation, and its function is altered as a consequence of ethanol intake. Luchtmann and co-workers analyzed ethanol-induced effects on the visuomotor system in 3 T magnetic resonance imaging at a BAC of 1.0‰^60^, demonstrating that this BAC appears to selectively disturb the connectivity between different brain areas such as the primary visual cortex, the supplementary motor area, and the left and right primary motor cortex^60^. Furthermore, they concluded that complex tasks requiring interaction or synchronization become affected even at moderate levels of alcohol. Though our given VR study has assessed much lower BAC levels, we may hypothesize that changes to brain connectivity may be similar but less pronounced in a dose-dependent manner. The study of Zheng *et al*. using functional magnetic resonance imaging could show consistent change in the functional activation and connectivity and of the superior frontal gyrus, cerebellum, hippocampal gyrus, left basal ganglia, and right internal capsule by ethanol^59^. This group concluded that the change in activity and connection may contribute to altered cognitive abilities and behavioral performance, underlining the findings from our study. Equally, alcohol-induced effects on the resting-state functional connectivity of the visual network appear to be selectively altered by acute alcohol consumption^61^. Though this effect may be less pronounced in low BAC levels, it may have implications for presence and user experience, as our data have shown. Future studies may show correlations between ethanol-induced impairments and changes in presence. Such observations could then reveal a part of physiological and neural pathways involved in the formation of presence.

It is important to understand the influence of drugs including ethanol on presence in VR, in light of the aforementioned effects. VR is no longer solely used for scientific purposes but has become a broadly available tool for entertainment and professional applications. To address the effects of ethanol a five-sided CAVE was used in which the participants took part in a geocaching tour whilst being influenced by ethanol with blood alcohol concentrations lower than 0.4‰. Surprisingly, the comparison of the ethanol group to the control group revealed that hardly any differences existed regarding the factors of the ITC-SOPI, SUS and the UEQ questionnaires, shown by the respective mean (median) scores and a comparably small standard deviation for all factors, indicating similarity. The high *P*-values for almost all factors and the η² showing no or only minute effects, emphasize our statement, despite the limitations of our study. Our hypothesis 1 claiming that ethanol consumption will affect presence and user experience therefore has to be rejected for the low-dose ethanol intake scenario investigated here. It can be concluded for the given sample and VR scenario that perceptive, cognitive and emotional aspects are similar and non-different between user experience and presence in the geocaching task between the control and ethanol groups.

In our previous published study^15^ using the same virtual scenario we could show that all presence factors correlated significantly with usability, and that most of them correlated with the user experience factors. Furthermore, we could show that presence and user experience factors are key indicators of validity when evaluating a product or system consisting of hard- and/or software in VR. Under the influence of ethanol, the participants were less influenced by presence in the assessment of usability and user experience. However, this correlation does not indicate a causal relationship between presence and user experience. Of the 28 possible correlations, only seven were found to be statistically significant. All of these seven correlations were moderate to high for the ethanol group (correlation coefficients larger than 0.3 and 0.5, respectively). In contrast, moderate or strong correlations were found for the control group with 18 of 28 possible correlations. The differing number of samples within each group is unlikely to explain this difference in correlations between user experience and presence. A close analysis of the *P*-values and correlation coefficients supports this statement. For those significant correlations, almost all *P*-values were very low and far from being borderline. Additionally, the differences in the correlation coefficients for significant correlations between the ethanol and control groups can be considered strong, e.g. for the correlation of ecological validity with stimulation the correlation coefficient is −0.03 in the ethanol group vs. 0.66 in the control group. These unexpected results indicate that a low dosage of ethanol might contribute to a disconnection between presence and user experience. These findings help to gain an important insight into subconscious and externally non-perceivable effects of ethanol consumption, whilst having a VR experience. The underlying central nervous system mechanisms causing this effect in VR are to date not completely known, and conclusions driven at this stage might be largely speculative. However, extensive research on ethanol as a pharmacological agent and psychoactive substance has been performed. It has been shown that ethanol does have manifold effects on the cortical regulation system^62,63^. Follow-up studies will need to clarify which of these effects, or which effect combination, may cause the disconnection between presence and user experience. Work that may give direction in this regard is that of Semmens-Wheeler *et al*.^64^, who found out that frontal lobe activity plays a critical role in responsiveness to hypnosis. One may hypothesize that a hypnotic experience can in some ways be seen as loosely related to a VR experience, as the participants’ perception system is targeted to induce a simulated experience to be perceived as real. Creating such a disconnection of presence and user experience may therefore be desirable to outbalance the existing shortcomings of VR systems in the application development for entertainment and professional scenarios. Vice versa, in games in a VR environment such disconnection might lead to a less enjoyable experience under the influence of low dose ethanol compared to the sober state. In professional applications, the supporting effect of presence on learnability may be removed when under the influence of confounders, leading to an inferior learning effect. Our hypothesis 2, stating that the consumption of ethanol has an influence on the correlations between presence and user experience, can therefore be accepted.

Comparison of the mean values between subjects from the fast and slow metabolizer groups revealed similar mean values and standard deviations for all presence and user experience factors with the only exceptions of negative effects and perspicuity. However, the effect size of the differences for negative effects and perspicuity were large^51^. As perspicuity describes how easy it is to understand, learn and use a product, one can conclude that the high metabolizers in our study understood the usage of the product more easily. The high metabolizers also suffered far less from negative effects of the VR experience. The effect sizes for the other three presence values showed no, or only a small effect, and also the *P*-values revealed no significant differences. This indicated that there is likely no difference between the fast and the slow metabolizers. Despite a clear difference for perspicuity, three other user experience factors and usability showed ambiguous results. Whilst the *P*-values for efficiency, dependability, stimulation and usability showed no significant differences, the η²-values demonstrated small or medium effect sizes. If these factors are influenced by ethanol breakdown kinetics remains unclear. Future research should concentrate on these factors. The remaining user experience factors, attractiveness and novelty, show no significant differences and no effect indicating that they are unaffected by ethanol breakdown kinetics. Further, there were no correlations between ethanol breakdown kinetics and presence or user experience. Our hypothesis 3 claiming that ethanol breakdown kinetics do influence presence and user experience and usability consequently can neither be rejected nor accepted on the basis of our data. Moreover, our data calls for more in-depth research on this topic.

Our study yielded a number of results with impact on VR in a psychological-medical-social context. Although a low dosage of ethanol barely caused differences in means, standard deviations and medians, there was a notable difference in the number of significant correlations (7 vs. 18). More specifically, this has implications regarding existing studies on the conceptual context of presence. One may speculate that under the influence of low dosages of ethanol the mode of decision-making is altered, or the way in which people report their experience in VR. Although this alteration does not necessarily change a decision or a rating itself, as the results for the user experience showed, it might have changed the rationale behind these decisions. This hypothesis could also initiate further investigation on how low dosage ethanol exposure is biasing the treatment results of classic VR involving therapies for phobias, stroke rehabilitation and PTSD. These methods should research if low dosage ethanol consumption has a strengthening, weakening or no influence on the therapy. The results of these studies should be considered when applying VR involving therapies. To emphasize this research and how ethanol may influence VR involving phobia treatments is especially relevant as alcohol-dependent patients often suffer from comorbid phobic disorders^36–38^. Therefore, it seems likely that some patients receiving phobia treatment, using VR technology, may be under the influence of ethanol. In light of our results future research should, in our opinion, concentrate on the effects different ethanol dosages and ethanol breakdown kinetics in alcohol-dependent patients have on presence and how it possibly affects phobia treatment results. In the context of VR drunk driving studies, our results indicate that low dose ethanol consumption does not necessarily change the perceived presence and user experience in a general group. This supports the credibility of the conducted research in the area of drunk driving studies using VR as an experimental environment. However, this applies only for low dosage ethanol consumption (<0.4‰). As far as higher ethanol dosages are concerned, commonly used in drunk driving studies, it remains unclear how presence and its connection to the typical measures used in this area are affected. Further research into the influence of different ethanol dosages on presence and its connection to the typical measures used in VR drunk driving studies, are recommended. Not only could such research improve future advancements in the aforementioned area, but it could also lead to a change in the interpretation of the existing results of conducted VR drunk driving studies. A further application area where the measured effects of our study could be relevant in future are VR-induced mental disorders, like PTSD, by playing VR video games. Currently the occurrence of such mental disorders is highly speculative and in fact no such cases have yet been reported. However, this might be reasoned by the unavailability of highly immersive VR battle games and that VR systems among consumers are still not widespread. However, such VR battle games and the required VR systems will very likely be available and widespread in the next years. Given it is most likely that the processes responsible for forming presence in the real world and VR are the same^18,19^, this may have strong implications for the onset of PTSD. For the authors it seems therefore highly likely that such disorders can also be induced through VR. If the consumers of VR battle games are feeling very present on the virtual battleground than why should they not be under the risk of suffering from PTSD especially when playing for hours? If this speculation turns out to be true, then also perception and cognition altering drugs like ethanol might play a role here, as they are an integral part of human culture^35^. Also, the successful treatment of PTSD patients with VR exposure therapy^1^ underline the psychological impact VR can have.

This given study presents a study protocol for researching the effects of ethanol in a VR environment, which could potentially serve as a starting point to further elucidate the effects of other substances on perception in a VR entertainment. However, to understand the effects of other substances on perception, a more elaborate study protocol is needed. It would require tasks that investigate more than spatial localization and somatosensory perception of goal-directed actions, such as a geocaching task. These tasks should include decision-making, risk-taking, craving assessment and the effect of drug-related cues which can be adapted from other studies in these fields^65–67^. This might especially be relevant for psychoactive or psychotropic drugs, sedatives, narcotics and gas anesthetics used in a hospital environment. In hospitals, today, patients with burn injuries or psychiatric disorders are already candidates to receive VR as a complementary treatment option. In a recent review on VR in mental health disorders, Freeman *et al*.^1^ also list VR treatment applications for psychosis like schizophrenia and substance disorders. Additionally, Dascal *et al*.^68^ and Hoffmann *et al*.^69,70^ demonstrate how pain ratings in burn-injury patients could be reduced effectively, distracting them using VR. However, it needs to be pointed out that drugs have differing effects on the central nervous system, which may also alter the decision-making process. Our study points out that there is a lack of questionnaires available to measure presence under the influence of ethanol. Therefore, the existing presence questionnaires should be improved in this direction or new ones developed.

A number of limitations need to be addressed for this study. First, only a limited number of participants were available due to the timeframe and the elaborate technical setup. Second, the VR scenario for geocaching has limitations regarding the mode of locomotion. Participants handled the locomotion with the Microsoft Kinect body tracking system differently, meaning that a few participants struggled more with controlling their movements inside the VR scenario, whilst the majority had no problems. This concerned four participants in the control group and one participant in the ethanol group who had issues controlling their movements, despite the tracking system working well. Second, the body tracking system worked differently between the participants, which is a known technical issue of the Microsoft Kinect sensor and its detection algorithms. One participant in each group suffered from bad body tracking. Using a more stable tracking sensor in future would probably resolve this issue. A qualitative analysis of the seven participants experiencing problems with the navigation gave no justification for an exclusion of their data sets. For each of the seven data sets it was evaluated if they solely caused the minimum or maximum values for each of the eleven factors of the dependent variables within their respective group. Only two of the seven data sets caused one minimum or maximum value. One other data set solely caused the minimum value for three of 11 factors. The remaining four data sets did not exclusively cause the minimum or maximum value for any of the 11 factors. We performed an analysis of the data without the seven data sets where navigation issues occurred, and the results were non-different (see supplement). Moreover, the Hawthorne effect might have influenced the outcomes of the study especially in respect to ethanol consumption, and also drug tolerance might be a possible confounder, and clear separation criteria were missing to separate slow from fast ethanol metabolizers. The performance at the task was not considered and the effect ethanol consumption had on it. Further, the timing of the last food intake might have played a role in ethanol absorption and breakdown kinetics, which was not substantiated in our present setting, though we tried to standardize this variable by providing a standard meal to each of the participants prior to the ethanol intake. Lastly, detailed ethanol consumption behavior was not obtained for the control group.

## Conclusions

This study researched the influence of low-dosage ethanol on presence and user experience in VR. Although the mean values of presence and user experience were similar in the ethanol and the control group, differences in the number of correlating presence and user experience factors were found. These differences indicated that central nervous system mechanisms supporting connections between presence and user experience might already become impaired at low dosages of ethanol. A comparison of the fast with the slow metabolizers showed ambiguous results. There were hardly any significant differences except for two of the 11 factors: perspicuity and negative effects. Future studies researching the effect of higher dosages of ethanol and of other drugs at differing dosages will therefore be of interest. Furthermore, it will be important to perform studies with different tasks and other virtual environments to see if similar results can be found, e.g. can we expect the same effects if people were playing a VR-game using a head mounted display?

## Electronic supplementary material

Supplement
