# Supplementary material for: Presence and User Experience in a Virtual Environment under the Influence of Ethanol: An Explorative Study
Source: Sci Rep. 2018 Apr 23;8:6407. doi: 10.1038/s41598-018-24453-5 (PMC5913276; doi:10.1038/s41598-018-24453-5)
Supplement: Supplementary file 1 — Supplement [file 41598_2018_24453_MOESM1_ESM.doc]

# Presence and User Experience in a Virtual Environment under the Influence of Ethanol: An Explorative Study

Mario Lorenz, Jennifer Brade, Lisa Diamond, Daniel Sjölie, Marc Busch, Manfred Tscheligi, Philipp Klimant, Christoph-E. Heyde, Niels Hammer

The following tables show the results of the statistical analysis without the seven data (five in the control group, two in the ethanol group) sets of the participants that experienced navigation issues.

Supplementary Table 1: Distribution of participant gender and professional occupation for the control and ethanol groups.

|  | Control Group | Ethanol Group |
| --- | --- | --- |
| Gender | Female: 15 (58%) | Female: 9 (43%) |
|  | Male: 11 (42 %) | Male: 12 (57%) |
| Professional | Students: 22 (85%) | Students: 10 (48%) |
| occupation | Other: 4 (15 %) | Other: 11 (52%) |

Supplementary Table 2: Distribution of participant age, results of the self-assessment in ability to read a map and distribution of previous contact with virtual reality systems (CAVE) and geocaching, for the control and ethanol groups including *P*-values (Mann-Whitney-U) testing for differences between the groups (green-highlighted fields are significant).

| Group | P-value | Control Group | Ethanol Group |
| --- | --- | --- | --- |
| Age (mean) | .75 | 25.9 | 24.3 |
|  |  | (SD=7.7) | (SD=4.0) |
| Ability to read a map (paper) | .87 | excellent=7 (27%) | excellent=7 (33%) |
|  |  | good=16 (62%) | good=9 (43%) |
| Ability to read a map (mobile) | .40 | excellent=9 (35%) | excellent=5 (24%) |
|  |  | good=14 (54%) | good=12 (57%) |
| Previous contact with VR-systems (yes) | .95 | 6 (23%) | 5 (24%) |
| Previous contact with geocaching (yes) | .046 | 9 (35%) | 2 (10%) |

Supplementary Table 3: Means, standard deviations (first row) and medians (second row) of the presence, usability and user experience factors for the control and ethanol groups with their *P*-values (Mann-Whitney-U) for significance testing and η²-values for effect sizes.

|  | P-value | η²-value | Control Group | Ethanol Group |
| --- | --- | --- | --- | --- |
| Ecological validity | .28 | .025 | 3.38 (SD=0.64) | 3.49 (SD=0.59) |
|  |  |  | 3.33 | 3.33 |
| Engagement | .92 | <.001 | 4.14 (SD=0.53) | 4.16 (SD=0.57) |
|  |  |  | 4.33 | 4.33 |
| Negative effects | .33 | .020 | 1.99 (SD=0.88) | 1.78 (SD=0.81) |
|  |  |  | 1.67 | 1.67 |
| Sense of physical space | .50 | .010 | 3.41 (SD=0.86) | 3.52 (SD=0.81) |
|  |  |  | 3.67 | 3.67 |
| Usability | .27 | .026 | 83.47 (SD=8.87) | 85.83 (SD=7.09) |
|  |  |  | 85.00 | 87.50 |
| Attractiveness | .71 | .003 | 1.69 (SD=0.65) | 1.55 (SD=0.76) |
|  |  |  | 1.67 | 1.67 |
| Dependability | .15 | .043 | 1.24 (SD=0.75) | 1.39 (SD=0.80) |
|  |  |  | 1.25 | 1.25 |
| Efficiency | .41 | .014 | 1.39 (SD=0.63) | 1.38 (SD=0.65) |
|  |  |  | 1.25 | 1.25 |
| Perspicuity | .48 | .011 | 1.96 (SD=0.76) | 2.02 (SD=0.89) |
|  |  |  | 2.25 | 2.25 |
| Novelty | .85 | .001 | 1.78 (SD=0.80) | 1.63 (SD=0.88) |
|  |  |  | 1.75 | 1.75 |
| Stimulation | .32 | .021 | 1.87 (SD=0.85) | 1.64 (SD=0.75) |
|  |  |  | 2.00 | 1.75 |

Supplementary Table 4: User experience factors and usability correlated (Spearman, two-tailed) with presence factors ("Ecological validity" and "Engagement") for the ethanol and control groups. With *P-*values (green-highlighted fields are significant).

|  | Ecological validity | | Engagement | |
| --- | --- | --- | --- | --- |
|  | Control Group | Ethanol Group | Control Group | Ethanol Group |
| Attractiveness | 0.61 (*P*<.001) | 0.50 (*P*=.02) | 0.80 (*P*<.001) | 0.34 (*P*=.14) |
| Dependability | 0.61 (*P=*.001) | 0.29 (*P*=.21) | 0.28 (*P*=.17) | -0.07 (*P*=.76) |
| Efficiency | 0.40 (*P*=.04) | 0.33 (*P*=.15) | 0.56 (*P*=.003) | 0.01 (*P*=0.94) |
| Perspicuity | 0.53 (*P*=.005) | 0.32 (*P*=.16) | 0.32 (*P*=.11) | 0.32 (*P*=.15) |
| Novelty | 0.24 (*P*=.23) | 0.50 (*P*=.02) | 0.26 (*P*=.21) | 0.28 (*P*=.23) |
| Stimulation | 0.42 (*P*=.03) | -0.01 (*P*=.98) | 0.49 (*P*=.01) | -0.01 (*P*=.96) |
| Usability | 0.42 (*P*=.02) | 0.21 (*P*=.37) | 0.56 (*P*=.02) | 0.39 (*P*=.08) |

Supplementary Table 5: User experience factors and usability correlated (Spearman, two-tailed) with presence factors "Negative effects" and "Sense of physical space") for the ethanol and control groups. With *P-*values (green-highlighted fields are significant).

|  | Negative effects | | Sense of physical space | |
| --- | --- | --- | --- | --- |
|  | Control Group | Ethanol Group | Control Group | Ethanol Group |
| Attractiveness | -0.11 (P=.59) | -0.19 (P=.41) | 0.63 (P<.001) | 0.48 (P=.03) |
| Dependability | -0.40 (P=.045) | -0.35 (P=.12) | 0.18 (P=.39) | 0.24 (P=.29) |
| Efficiency | -0.06 (P=.78) | -0.33 (P=.15) | 0.49 (P=. 01) | 0.26 (P=.25) |
| Perspicuity | -0.41 (P=.04) | -0.65 (P=.001) | 0.31 (P=.12) | 0.24 (P=.30) |
| Novelty | 0.31 (P=.13) | -0.16 (P=.49) | 0.39(P=.051) | 0.66 (P=.001) |
| Stimulation | 0.10 (P=.64) | -0.36 (P=.11) | 0.43 (P=.03) | 0.25 (P=.27) |
| Usability | -0.54 (P=.005) | -0.72 (P<.001) | 0.31 (P=.12) | 0.30 (P=.20) |

Supplementary Table 6: Means, standard deviations (first row) and medians (second row) of the presence, usability and user experience factors for the fast and slow metabolisers and their *P*-values (Mann-Whitney-U) for significance testing (green-highlighted fields are significant) and η²-values for effect size.

|  | P-value | η²-value | Slow metabolisers | Fast metabolisers |
| --- | --- | --- | --- | --- |
| Ecological validity | .64 | .010 | 3.42 (SD=0.63) | 3.57 (SD=0.57) |
|  |  |  | 3.33 | 3.50 |
| Engagement | .39 | .035 | 4.03 (SD=0.67) | 4.30 (SD=0.43) |
|  |  |  | 4.00 | 4.33 |
| Negative effects | .07 | .154 | 2.12 (SD=0.93) | 1.40 (SD=0.41) |
|  |  |  | 1.67 | 1.33 |
| Sense of physical space | .41 | .033 | 3.33 (SD=1.02) | 3.73 (SD=0.47) |
|  |  |  | 3.33 | 3.67 |
| Usability | .55 | .017 | 84.55 (SD=8.05) | 87.25(SD=5.95) |
|  |  |  | 85.00 | 88.75 |
| Attractiveness | .57 | .015 | 1.45 (SD=0.72) | 1.65 (SD=0.83) |
|  |  |  | 1.50 | 1.67 |
| Dependability | .12 | .116 | 1.11 (SD=0.84) | 1.70 (SD=0.67) |
|  |  |  | 1.25 | 1.88 |
| Efficiency | .28 | .055 | 1.20 (SD=0.72) | 1.58 (SD=0.53) |
|  |  |  | 1.25 | 1.50 |
| Perspicuity | .01 | .316 | 1.57 (SD=0.94) | 2.53 (SD=0.51) |
|  |  |  | 1.75 | 2.50 |
| Novelty | 0.86 | .001 | 1.66 (SD=0.90) | 1.60 (SD=0.91) |
|  |  |  | 2.00 | 1.75 |
| Stimulation | .50 | .022 | 1.48 (SD=0.84) | 1.83 (SD=0.62) |
|  |  |  | 1.50 | 1.75 |

The following table shows the Cronbachs‘ alpha values.

Supplementary Table 7: Values of Cronbachs’ alpha for the scales of the ITC-SOPI, SUS and UEQ

|  | **Scale** | **CA** |
| --- | --- | --- |
| **ITC-SOPI** | Sense of physical space | 0.85 |
|  | Engagement | 0.62 |
|  | Ecological validity | 0.55 |
|  | Negative effects | 0.69 |
| **SUS** | Usability | 0.68 |
| **UEQ** | Attractiveness | 0.81 |
|  | Perspicuity | 0.51 |
|  | Efficiency | 0.27 |
|  | Dependability | 0.50 |
|  | Stimulation | 0.58 |
|  | Novelty | 0.63 |
